# In Search of New Drugs: Elucidating the Activity of Structurally Similar Potential Antibiotics Using Molecular Modelling

**DOI:** 10.3390/molecules30142920

**Published:** 2025-07-10

**Authors:** Natalina Makieieva, Teobald Kupka, Piotr Lodowski, Radosław Balwierz, Katarzyna Kasperkiewicz, Adam Byrski, Roksolana Konechna, Vira Lubenets

**Affiliations:** 1 Faculty of Chemistry and Pharmacy, University of Opole, Oleska 48, 45-052 Opole, Poland; radoslaw.balwierz@uni.opole.pl; 2 Institute of Chemistry, University of Silesia in Katowice, Szkolna 9, 40-006 Katowice, Poland; piotr.lodowski@us.edu.pl; 3 Faculty of Natural Sciences, Institute of Biology, Biotechnology and Environmental Protection, University of Silesia in Katowice, Jagiellonska 28, 40-032 Katowice, Poland; katarzyna.kasperkiewicz@us.edu.pl; 4 Institute of Metallurgy and Materials Science, Polish Academy of Sciences, Reymonta 25, 30-059 Cracow, Poland; a.byrski@imim.pl; 5 Department of Technology of Biologically Active Substances, Pharmacy and Biotechnology of Lviv Polytechnic National University, S. Bandery 12, 79000 Lviv, Ukraine; roksolana.t.konechna@lpnu.ua (R.K.); vira.i.lubenets@lpnu.ua (V.L.)

**Keywords:** S-containing antibiotics, DFT, NBO, structure-activity relationship

## Abstract

The global problem of antibiotic resistance leads to the necessity for drug improvement and discovery. Natural and synthetic sulfur-containing compounds have been known as antibiotics for many years. In the current study, we demonstrated an antibacterial activity of three new thiosulfonates: S-ethyl 4-aminobenzene-1-sulfonothioate (1), S-methyl 4-acetamidobenzene-1-sulfonothioate (2), and S-ethyl 4-acetamidobenzene-1-sulfonothioate (3). Their activities were studied on two model Gram-positive and Gram-negative bacteria strains: *Staphylococcus aureus* ATTC 6538P and *Escherichia coli* ATTC 8739, respectively. According to the literature data, we proposed a general mechanism of 1−3 biochemical actions. To analyze its feasibility, theoretical studies using density functional theory (DFT) were performed. The obtained results demonstrate a direct correlation between some NBO parameters and the S-S bond energy of 1−3 with their activity against both studied bacterial strains. The obtained results could be helpful for future biomedical studies on the analyzed compounds and promote the further design of new S-containing antibiotics.

## 1. Introduction

Antimicrobial resistance (AMR) has become one of the key healthcare issues in recent years. This condition occurs when populations of pathogenic microorganisms (bacteria, fungi, protozoa and viruses) in the host organism are resistant to the toxic effects of drugs. The reason for this phenomenon is primarily the excessive use of antibiotics without necessity, namely self-medication, erroneous prescription of medication or the demand for medication from medical personnel. Other factors include the excessive use of antibiotics in veterinary and agronomy, which leads to the accumulation of drugs in animal and plant foods consumed. In addition, insufficient purification of wastewater from antibiotics and their degradation by-products leads to the accumulation of drugs in the environment and their subsequent entry into plant, animal and human organisms [1,2,3]. Mortality due to human AMR is gradually increasing on the global stage. In 2019, 1.2 million deaths were registered, and according to World Health Organization (WHO) predictions, without modernization of antimicrobial therapies, we should expect about 10 million deaths per year [1]. This negative trend makes it necessary to analyze the existing antibiotic market in relation to the actual drugs’ effectiveness and to develop new medicines with an improved pharmacological profile.

**Figure 1:**
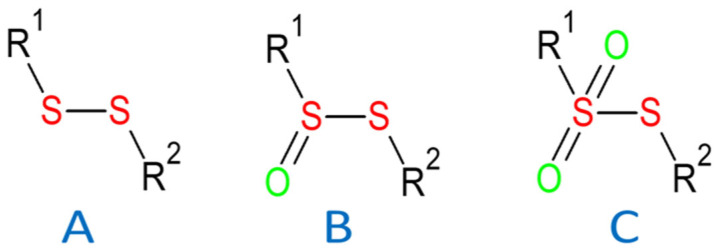
Schematic formulas of (**A**) disulfides, (**B**) thiosulfinates and (**C**) thiosulfonates antibiotics [4].

One of the promising groups of antibiotics is -S-S- structural motive-containing organic compounds, namely disulfides, thiosulfinates and thiosulfonates (see the schematic formulas of each group in Figure 1).

**Figure 2:**
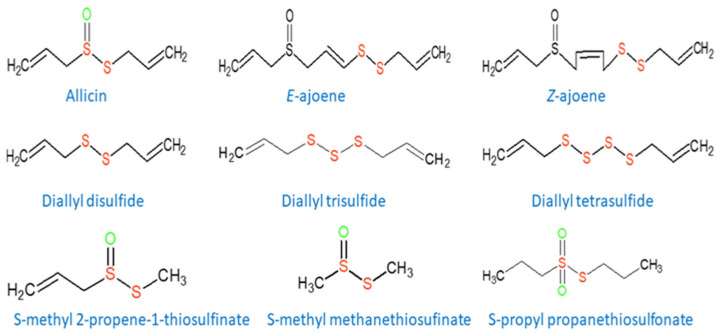
Structures of some organosulfur metabolites of the *Allium* genus plants with antimicrobial activity.

The effectiveness of these compounds in the treatment of diseases of bacterial, fungal and viral origin has been known for a long time. The first representatives used in medical practice were metabolites of some *Allium* genus plants (see Figure 2) [4,5,6].

The activity of these compounds has been tested against a number of viruses [7], fungi [8], and Gram-positive and Gram-negative bacteria [5]. However, the development of microorganisms’ resistance to the action of traditional antibiotics leads to the limited spectrum of *Allium* metabolites use. For this reason, synergistic studies of the drugs’ action mechanisms and resistance to them are being conducted. A number of works describing the action of known antibiotics, the adaptation of microorganisms to them and the proposal of alternative medicines with a modified spectrum of action and more efficient destruction of the pathogen are presented [9,10,11,12,13]. For example, recent literature data suggest that the key mechanism of bacterial adaptation is modification of genetic material [14]. This leads to a change in the composition or targeted destruction of the cell membrane and, as a consequence, a decrease in drug permeability [14]. Another mechanism is a metabolic activity reduction leading to a decrease in the expression of antibiotic molecular targets. Bacterial metabolism modification could also provide new pathway formation, which leads to the modification or inactivation of drugs. Finally, an exchange of genetic material within the cells in biofilm provides the acquisition of resistance mechanisms in the colony [14]. To block the protective mechanisms and increase the permeability of the drug into the bacterial cell, the literature suggests the use of new classes of compounds: antibacterial proteins [15,16,17,18], metal nanoparticles [19,20], polymers [21,22,23] and others [24,25,26]. An increase in antibiotic activity has been observed when they are introduced into drug delivery systems, amplifying penetration into the bacterial cell [27,28,29]. Particular attention has been paid to the development of new synthetic compounds with an improved clinical profile. For example, D-modification of the amino acid sequence increases the antibacterial activity, safety and stability of the antibacterial proteins [30]. A strong structure–activity correlation was studied for a series of synthetic myrtucommulone analogues as potential inhibitors of DNA gyrase and topoisomerase IV [25]. Relatively little is known about the structural modification effect on the activity of organosulfur antibiotics, especially thiosulfonates. Only a few systematic studies present an attempt to establish the structure–activity relationship [31,32,33,34]. In the case of thiosulfonates, this is of particular importance, since in addition to their antibacterial properties, their anticancer potential has been noted [31,35,36,37].

**Figure 3:**
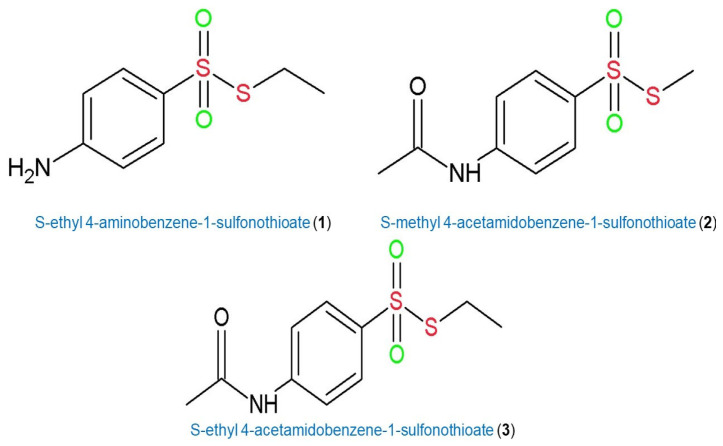
Structures of thiosulfonates **1**−**3**.

The aim of our work was to expand the existing knowledge about the influence of structure on the antibacterial activity of thiosulfonates. We present data on the activity of three new derivatives of the thiosulfonate group: S-ethyl 4-aminobenzene-1-sulfonothioate (**1**), S-methyl 4-acetamidobenzene-1-sulfonothioate (**2**) and S-ethyl 4-acetamidobenzene-1-sulfonothioate (**3**) (see Figure 3). Microbiological tests were carried out on two model bacteria: Gram-positive *Staphylococcus aureus* ATTC 6538P and Gram-negative *Escherichia coli* ATTC 8739. In order to explain the different activities of structurally similar compounds, a systematic theoretical study was carried out.

## 2. Results and Discussion

**Figure 4:**
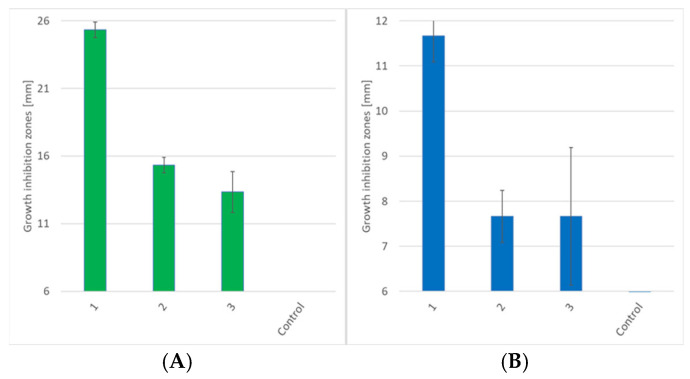
Antibacterial activity of thiosulfonates **1**−**3** compared with the control sample against (**A**) Gram-positive *S. aureus* and (**B**) Gram-negative *E. coli* strains.

Based on the obtained results (see Figure 4), it can be concluded that compound **1** has the highest activity against both Gram-positive and Gram-negative bacteria. It is also worth noting that substances **2** and **3** had similar activity against both *S. aureus* and *E. coli*. Derivative **1** significantly differed structurally from **2** and **3** (according to Figure 3, its amine end was not modified to amide), while **2** and **3** differed only in the length of the alkyl chain at the S-end.

**Figure 5:**
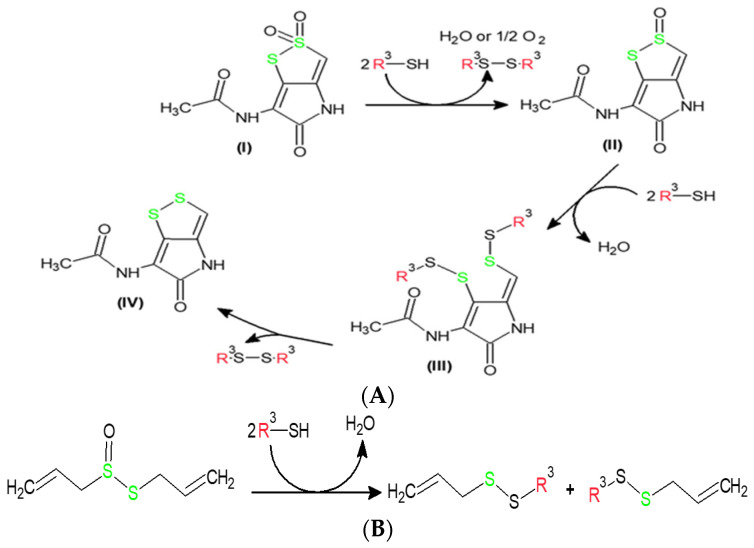
Mechanisms of reaction of (**A**) homolycin and (**B**) allicin with glutathione or cysteine residues (R^3^-SH) [38,39].

**Figure 6:**
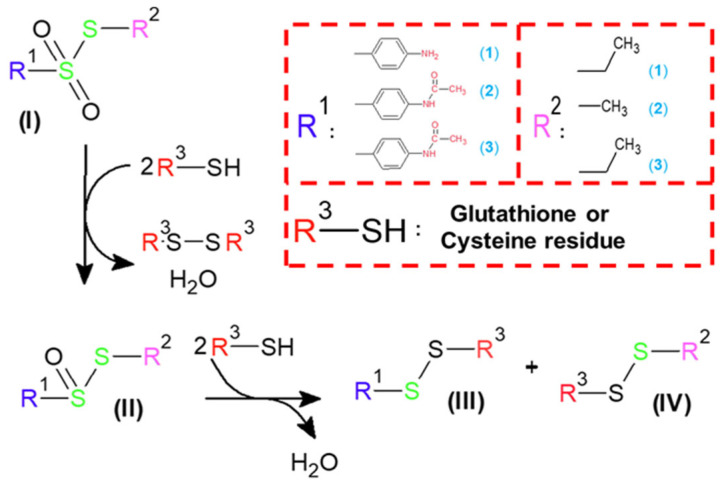
Proposed general mechanism of compounds **1**−**3** antibacterial action.

The mechanism of thiosulfonates **1**−**3** antibacterial action is not described in the scientific literature. Moreover, limited data also describe the molecular mechanisms of other thiosulfonate antibiotic action [31,32,33,34]. However, some systematic works present the mechanisms of action of allicin and homolycin [38,39] (see Figure 5). It was described in [38,39] that both antibiotic molecular targets in bacterial cells are glutathione and cysteine residues of different peptide structures (enzymes, membrane peptides, etc.). As can be seen from Figure 5A, homolycin is a cyclic thiosulfonate. As a result of the first stage of its interactions with glutathione (GSH) or cysteine residues (Cys) of protein structures (in Figure 5, GSH/Cys are presented as R^3^-SH), it is reduced to cyclic thiosulfinate. This intermediate has a leading structural motif O=S-S-, similar to allicin. According to their structural similarity, it was supposed that further processing of reduction occurs in a similar way as in the case of allicin (Figure 5B). In contrast to **1**−**3** thiosulfonates, due to homolycin’s cyclic structure, there is no destruction of the drug molecule, but the closure of the disulfide bridge with simultaneous oxidation of GSH/Cys (R^3^S-SR^3^, Figure 5A). Based on the literature data, a theoretical mechanism of thiosulfonates **1**−**3**’s antibacterial action is presented in Figure 6. To analyze the feasibility of this general mechanism, further systematic molecular modelling was performed.

During molecular modelling, certain parameters were calculated. First, the NBO charges and orbitals [40] were analyzed. According to the proposed reaction mechanism (see Figure 6), during biochemical transformations, the oxidized sulfur atom in the structure of **1**−**3** is subject to stepwise reduction. Therefore, the distribution of partial charges in the potential antibiotic molecules can serve as a key factor in the reaction rate and, consequently, biological activity. As can be seen from Figure 6, the -SO_2_-S- structural moiety is subject to modification to -SO-S- and further cleavage of the -S-S- bond. Therefore, it is assumed that an increase in the polarity of the -S=O bond will have a key effect in initiating the first and second stages of the reaction of **1**−**3** with biomolecules. As can be seen from Table 1, bond polarization decreases with decreasing antibacterial activity in **1**−**3**. In addition, it can be noted that the difference in sulfur and two oxygen atoms NBO charges increases with decreasing activity. This indicates a decrease in the electron density on the oxygen atoms and, as a consequence, their lower ability to nucleophilic attack. This leads to a slowdown in the first stage of biochemical transformations of thiosulfonates **2** and **3**. It is worth mentioning that for all analyzed thiosulfonates **1**−**3,** a similar trend of NBO charges and orbital charges is observed in vacuum and in water (PCM). It can be noted that in water, the difference between sulfur and the two oxygen atoms NBO charges is not as significant as in vacuum. As can be seen from Table 1, in a polar environment, the negative charge on the oxygen atoms increases significantly. This can be explained by the effect of additional polarization of the environment, as well as by the improved prediction and stabilization of partial charges in the polar solvent. Similar values of the difference between sulfur and two oxygen atoms NBO charges in compounds **2** and **3** in a polar environment could be explained by their similar antibacterial activity (see Figure 4).

Analyzing the NBO orbitals (see Table 1), a similar growth of the -S=O1 bond polarization with antibacterial activity increase is observed both in vacuum and in water (PCM). After the first stage of reduction to transition products with the -SO-S- moiety, a similar dependence of the distribution of partial charges and the contribution of the NBO orbitals on biological activity is observed. As can be seen from Table 2, the smallest ΔNBO(S-O) value is obtained for the most active thiosulfinate **1**. Moreover, the oxygen atom in **1** has the largest negative charge and, therefore, the largest electron density. Therefore, as in the case of thiosulfonate **1**, the highest ability of its thiosulfinate to initiate a nucleophilic attack in reactions with glutathione or cysteine residues is expected. In the case of the NBO orbitals, the maximal polarization of the -S=O bond is also observed for the compound **1** transition product, which further indicates its highest reactivity (see Table 2). Moreover, the thiosulfinates’ NBO parameters calculated both in vacuum and in water correlate with the total activity of **1**−**3** (see Figure 4 and Table 2).

Analyzing the proposed reaction mechanism, it can be noted that the final stage involves the S-S bond cleavage in the transition products of **1**−**3**. Based on this, it can be assumed that the activity of potential antibiotics will correlate with the S-S bond energy. As can be seen from Table 3, the lowest value of the S-S bond dissociation energy was observed in the case of compound **1** thiosulfinate. The energy gradually increased for **2** and **3**, with a decrease in antibacterial activity. It is worth noting that this correlation was obtained both in the case of the dissociation energy with and without the ZPE correction in vacuum and in water (PCM). Based on this, it can be assumed that the S-S bond energy plays a significant role in the process of biochemical reactions of **1**−**3** with glutathione and cysteine residues. According to the II part of the proposed general mechanism of biochemical action (see Figure 6), it can be suspected that the weakening of S-S leads to an increase in the reactivity of compound **1** toward molecular targets (glutathione and cysteine residues). This may lead to acceleration and enhancement of the antibacterial action of compound **1** against Gram-negative and Gram-positive bacteria. However, this assumption requires additional confirmation by systematic theoretical and experimental studies.

To summarize, a direct correlation between the NBO charges of oxygen atoms in the S=O motif and the polarization of this bond, as well as the energy of the S-S bond dissociation with the compounds **1**−**3,** antibacterial activity was observed. It is worth noting that the correlation was obtained in both the gas phase and water (PCM) calculations. The results partially confirm the proposed general mechanism of biochemical transformations of thiosulfonates **1**−**3** (Figure 6) in Gram-positive and Gram-negative bacteria cells. It can be stated that although the analyzed compounds are structurally similar, they exhibit different abilities for nucleophilic attacks on molecular targets in the bacteria cells. However, a detailed description of compounds **1**−**3** transformation mechanisms at the molecular level requires additional theoretical and experimental studies. They will be presented in further works.

## 3. Materials and Methods

### 3.1. Synthesis and Purification

The procedure of the synthesis of all analyzed thiosulfonates **1**–**3** has been presented previously [41,42]. The purity of the obtained samples was determined based on data from a number of analytical methods. Reaction product purity was determined using thin-layer chromatography (TLC) on “Silufol UV 254” plates. The melting points were determined without correction in open capillary tubes. Liquid chromatograph-mass spectrometry (LC-MS) spectra were also recorded using an Agilent 110\DAD\HSD\VLG 119,562 apparatus (Agilent, Headquarters, Santa Clara, CA, USA). Ionization by electrospray was performed under atmospheric pressure (70 eV). Elemental analysis of the samples was performed using the Perkin Elmer CHN-Analyzer series 2400 (Perkin Elmer, Waltham, MA, USA). Complex spectrometric analysis was also performed. IR spectra were measured using a Thermo Nicolet spectrometer (Nexus Analytics, Madison, WI, USA) (see Figures S3 and S6). The ^1^H and ^13^C NMR spectra were recorded using an Ultrashield Bruker spectrometer (400 MHz) (Bruker Optik GmbH, Ettlingen, Germany) in DMSO-d6. The chemical shifts were reported relative to TMS (see Figure S1, S2, S4 and S5).

### 3.2. Antimicrobial Activity

#### 3.2.1. Bacterial Strains and Tested Compounds Solutions

Antibacterial activity of **1**−**3** was tested on two bacterial strains: *Escherichia coli* ATTC 8739 and *Staphylococcus aureus* ATTC 6538P as model Gram-positive and Gram-negative bacteria, respectively. The strains were taken from the collection of the Institute of Biology, Biotechnology and Environmental Protection (University of Silesia in Katowice). Prior to tests, microorganisms were cultivated overnight at 36  ±  1 °C using Nutrient broth (Merck Millipore, Burlington, MA, USA; for *E. coli*) and Tryptic soy broth (Merck Millipore, Burlington, MA, USA; for *S. aureus*) media, previously sterilized in an autoclave (15 min, 121 °C). Each of the **1**−**3** tested compounds was dissolved in phosphate-buffered saline (PBS) at pH 7.4. The concentration of each final solution was 100 mg/mL. This concentration was chosen because the aim of the study was to determine compounds’ potential antibacterial properties without minimum inhibitory concentration measurements.

#### 3.2.2. Disk Diffusion Method

The antimicrobial activity was analyzed using the disc diffusion test on Mueller–Hinton agar plates. The inoculum was prepared as a suspension with a turbidity of 0.5 on the McFarland scale (approximately optical density (OD) at 600  nm is 0.06), which was achieved by diluting overnight cultures in PBS. The bacteria were seeded on the Mueller–Hinton agar using the turf method. Sterile paper discs of 6 mm diameter were partially immersed in the tested **1**−**3** compounds solutions and control (PBS) and subsequently applied to the plates in three replicates. The plates were inverted and incubated for 24 h at 37 °C in a humidified (~90%) atmosphere. The diameter of the bright zones was then measured as a proportional value to the sensitivity of the tested strain to a given compound. The results are presented as the mean of three replicates.

#### 3.2.3. Statistical Analysis

A parametric analysis of variance ANOVA was used to compare microbial inhibition zone values. For post hoc multiple comparisons, Fisher’s LSD test was used. In all analyses and statistical tests performed, a significance level of α = 0.05 was adopted.

### 3.3. Theoretical Studies

All calculations were performed using Gaussian 16 C.01 software [43]. The first step was the structural optimization. To analyze the influence of the environment polarity on the chemical activity of **1**−**3**, optimization and all theoretical parameters were calculated in the gas phase and water using the Polarizable Continuum Model (PCM) [44]. The structure optimization was performed for neutral forms of **1**−**3** thiosulfonates and thiosulfinates (geometries are presented in Tables S1–S4). The criterion for obtaining the energetically equilibrium structure was the absence of negative values of harmonic vibrational frequencies. All calculations were performed using the hybrid density functional of Becke-Lee-Yang-Parr B3LYP [45,46,47] with Grimm’s empirical correction for dispersion (D3BJ) [48]. This method demonstrated acceptable accuracy in calculating the structural, spectroscopic and energy parameters of small and medium-sized molecules [49,50]. In order to obtain all structural, energy and NBO values with satisfactory accuracy, the Pople’s 6-311++G** basis set was used [51]. The S-S bond dissociation was analyzed as homolytic fission. This mechanism was used based on the analysis of the NBO orbitals, which indicate the covalence of the S-S moiety in all analyzed thiosulfinates (see Table A1 in the Appendix A). Bond dissociation energies in kcal/mol were calculated according to the following formula:

(1) $$∆E_{R_{1}S-SR_{2}}=\left(E_{R_{1}S}+E_{R_{2}S}-E_{R_{1}S-SR_{2}}\right)\;*\;627.509,$$

where $∆E_{R_{1}S-{SR}_{2}}$ is electronic energy without or with zero-point energy (ZPE) of S-S bond dissociation, while E are electronic energies without or with zero-point energies (ZPE) of the studied compound or its fragments.

## 4. Conclusions

In this work, a comparative characteristic of the biological activity of three new thiosulfinates is presented. The antibacterial potential of this group of compounds is described in the literature. However, their mechanism of action at the molecular level has not been studied in detail. In addition, very little data are presented on the dependence of thiosulfonate activity on structural modification. In this work, the activity of three structurally similar thiosulfonates, S-ethyl 4-aminobenzene-1-sulfonothioate (**1**), S-methyl 4-acetamidobenzene-1-sulfonothioate (**2**) and S-ethyl 4-acetamidobenzene-1-sulfonothioate (**3**) against *Escherichia coli* ATTC 8739 as a model Gram-negative bacteria and *Staphylococcus aureus* ATTC 6538P as a model Gram-positive bacteria, was analyzed. A significantly different high activity of compound **1** against both bacterial species is noted. At the same time, it is noted that compounds **2** and **3**’s activities were quite similar. To explain the relationship between structure and activity, a potential general mechanism of antibacterial activity was proposed based on the available literature. To determine its feasibility, molecular modelling of some parameters of compounds **1**−**3** was performed. A direct correlation between the charge on the oxygen atom and the polarization of the -S=O bond with the overall activity of compounds **1**−**3** was noted. In addition, a connection between the energy of the S-S bond of the **1**−**3** transition products and the overall activity against *E. coli* and *S. aureus* was noted. Theoretical data demonstrate the possibility of the proposed general mechanism. However, additional experimental and theoretical studies are needed to describe the mechanism of antibacterial action at the molecular level in detail.

## Figures and Tables

**Table 1:** NBO charges and orbitals of -SO_2_- structural motif in thiosulfonates **1**−**3**, calculated in the gas phase and water (PCM).

Compound	NBO Charges–Gas Phase				NBO Charges–Water (PCM)			
	S(O_2_)	O1	O2	Δ(S-2O)	S(O_2_)	O1	O2	Δ(S-2O)
1	1.990	−0.903	−0.892	0.195	1.914	−0.935	−0.939	0.040
2	1.986	−0.895	−0.893	0.198	1.909	−0.927	−0.933	0.049
3	2.031	−0.898	−0.890	0.243	1.909	−0.928	−0.932	0.049
**Compound**	**NBO Orbitals–Gas Phase, %**				**NBO Orbitals–Water (PCM), %**			
	**S(O_2_)**	**O1**	**S(O_2_)**	**O2**	**S(O_2_)**	**O1**	**S(O_2_)**	**O2**
1	35.91	64.09	36.25	63.75	35.74	64.26	35.95	64.05
2	35.94	64.06	36.25	63.75	35.78	64.22	36.00	64.00
3	36.00	64.00	36.25	63.75	35.81	64.19	36.01	63.99

**Table 2:** NBO charges and orbitals of the -SO- structural motif in thiosulfinates **1**−**3**, calculated in the gas phase and water (PCM).

Compound	NBO Charges–Gas Phase			NBO Charges–Water (PCM)		
	S(O)	O	Δ(S-O)	S(O)	O	Δ(S-O)
1	1.097	−0.906	0.191	1.100	−0.959	0.141
2	1.095	−0.900	0.195	1.109	−0.965	0.144
3	1.108	−0.897	0.211	1.103	−0.957	0.146
**Compound**	**NBO Orbitals–Gas Phase, %**			**NBO Orbitals–Water (PCM), %**		
	**S(O)**	**O**		**S(O)**	**O**	
1	36.49	63.51		36.08	63.92	
2	36.54	63.46		36.14	63.86	
3	36.56	63.44		36.17	63.83	

**Table 3:** Dissociation energies (kcal/mol) of the S-S bond of **1**−**3** thiosulfinates without and with ZPE value calculated in vacuum and water (PCM).

Compound	Gas phase		Water (PCM)	
	E	E + ZPE	E	E + ZPE
1	−30.5566	−27.8834	−31.4194	−28.7205
2	−34.7697	−31.6691	−33.9137	−31.0096
3	−35.1643	−32.3023	−34.2955	−31.4583

## Data Availability

Data is contained within the article.

## Supplementary Materials

The following supporting information can be downloaded at: https://www.mdpi.com/article/10.3390/molecules30142920/s1.

## Appendix A

**Table A1:** NBO orbitals of S-S bonds in thiosulfinates **1**−**3**, calculated in the gas phase and water (PCM).

Compound	NBO Orbitals–Gas phase, %		NBO Orbitals–Water (PCM), %	
	S(O)	S	S(O)	S
1	50.14	49.86	50.03	49.97
2	49.79	50.21	50.20	49.80
3	49.30	50.70	49.59	50.41
