# Supplementary material for: In Search of New Drugs: Elucidating the Activity of Structurally Similar Potential Antibiotics Using Molecular Modelling
Source: Molecules. 2025 Jul 10;30(14):2920. doi: 10.3390/molecules30142920 (PMC12300642; doi:10.3390/molecules30142920)
Supplement: Supplementary file 1 [file molecules-30-02920-s001.zip › molecules-3720591-supplementary resubmited.pdf]

SUPPORTING INFORMATION FOR

In Search of New Drugs: Elucidating the Activity of Structurally Similar Potential Antibiotics Using Molecular Modelling

Natalina Makieieva*, Teobald Kupka*, Piotr Lodowski, Radosław Balwierz, Katarzyna Kasperkiewicz, Adam Byrski, Roksolana Konechna and Vira Lubenets

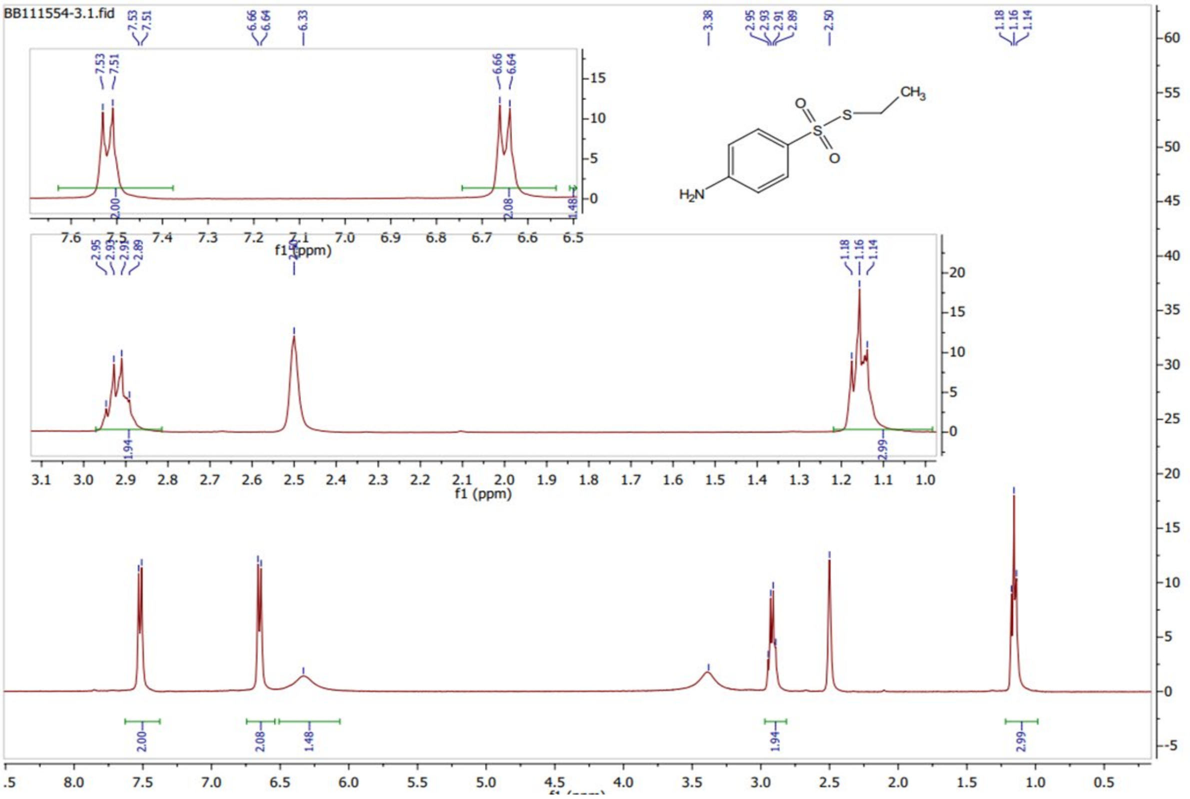

Figure S1. ^{1}H NMR spectrum of S-ethyl 4-aminobenzene-1-sulfonothioate (1) in DMSO-d6

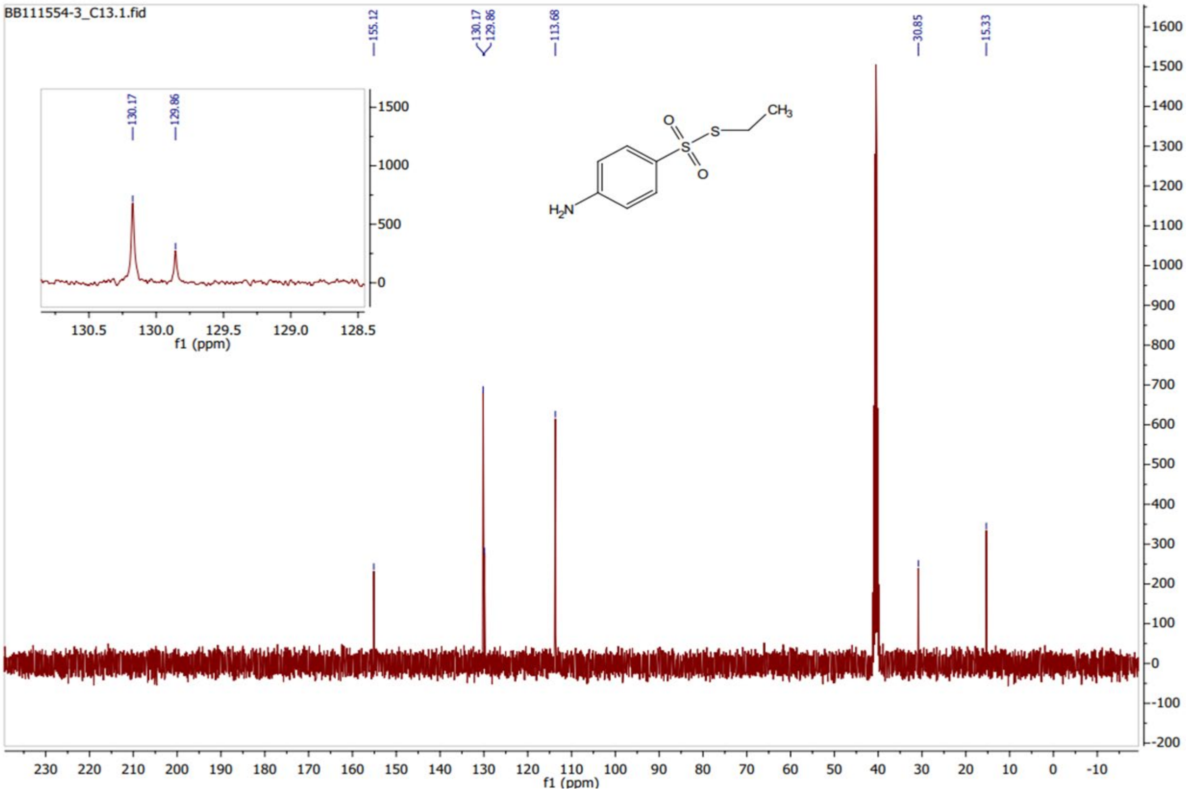

Figure S2. ^{13}C NMR spectrum of S-ethyl 4-aminobenzene-1-sulfonothioate (1) in DMSO-d6

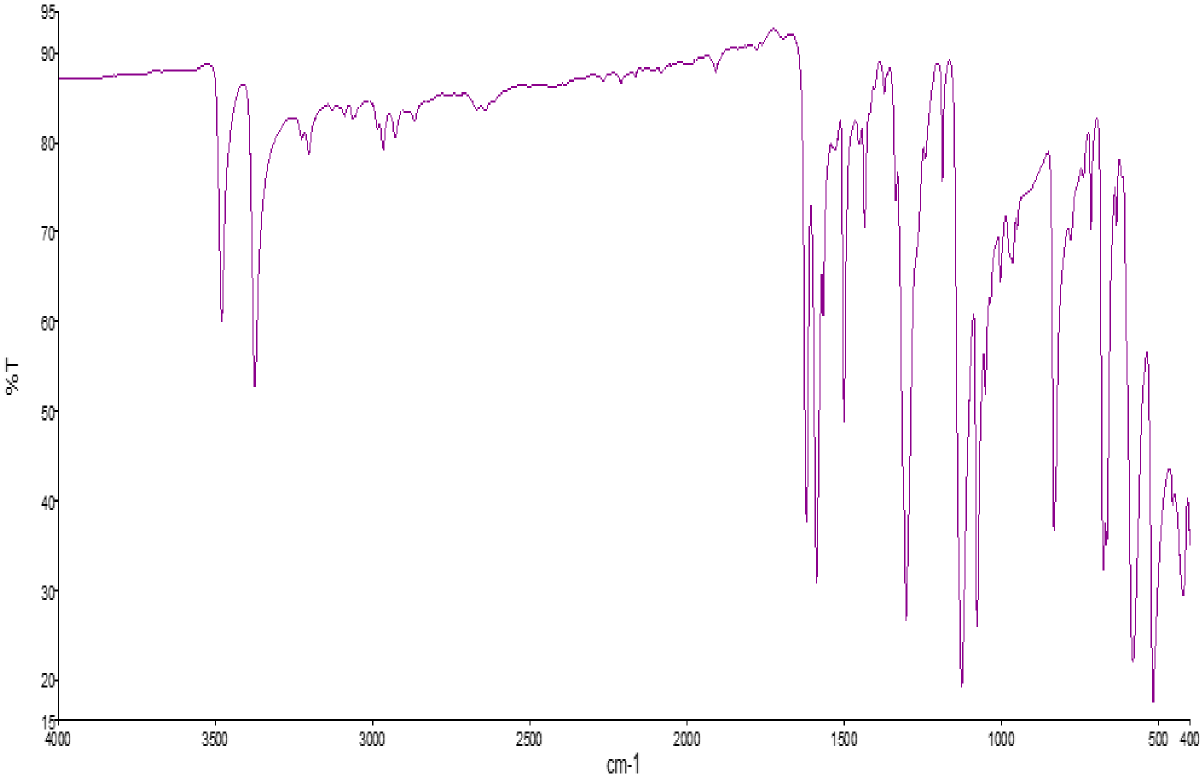

Figure S3. IR spectrum of S-ethyl 4-aminobenzene-1-sulfonothioate (1)

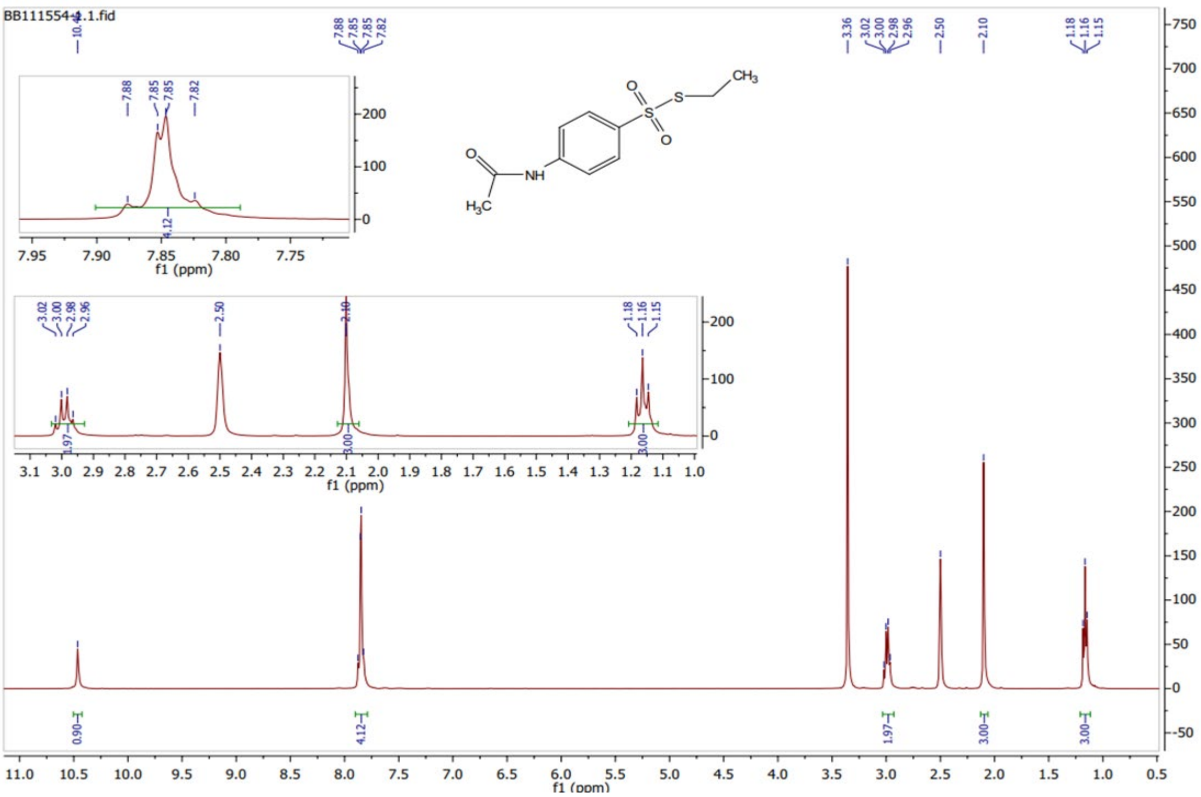

Figure S4. ^{1}H NMR spectrum of S-ethyl 4-acetamidobenzene-1-sulfonothioate (3) in DMSO-d6

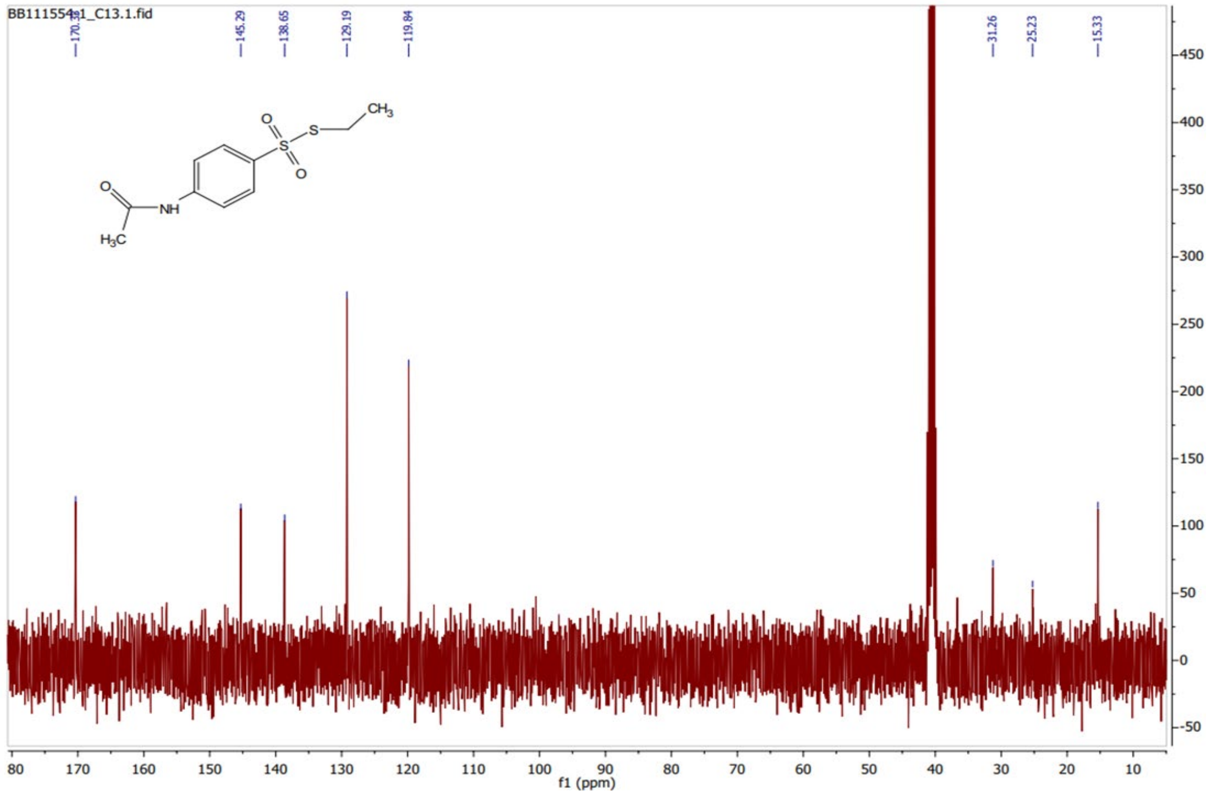

Figure S5. ^{13}C NMR spectrum of S-ethyl 4-acetamidobenzene-1-sulfonothioate (3) in DMSO-d6

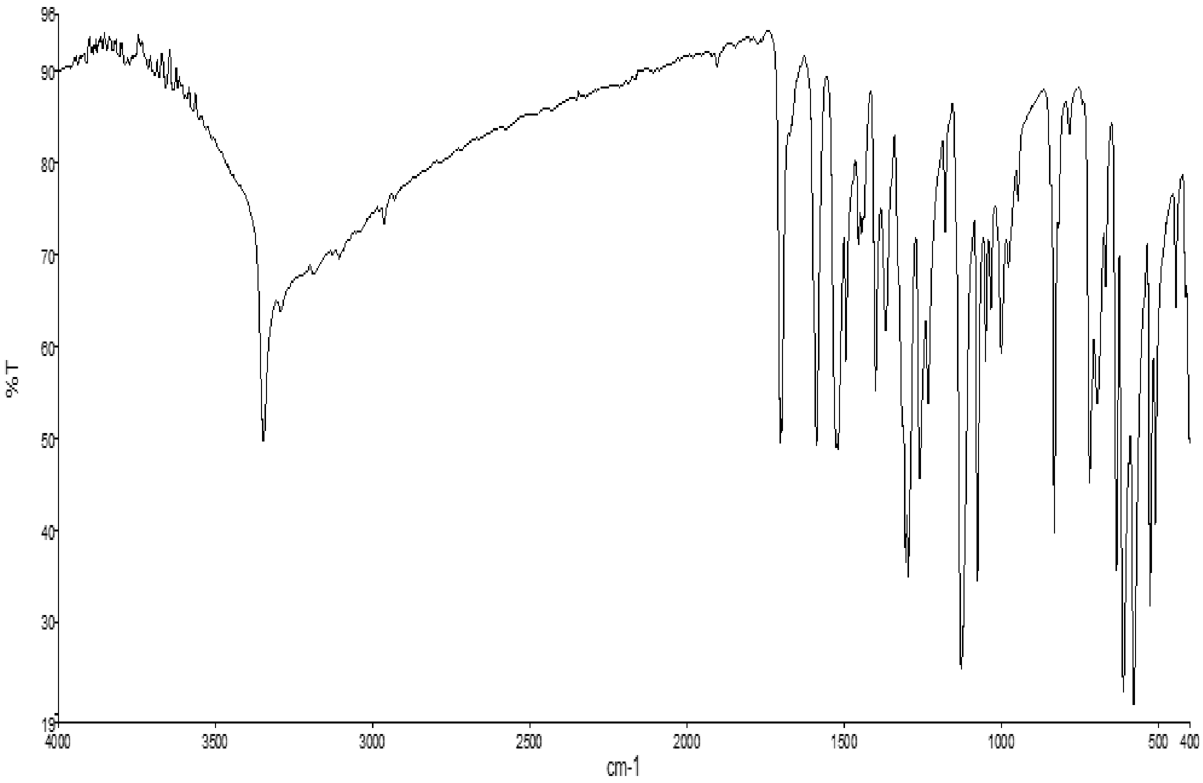

Figure S6. IR spectrum of S-ethyl 4-acetamidobenzene-1-sulfonothioate (3)

Table S1. Cartesian cordinates of thiosulfonates **1-3** calculated optimised at B3LYPGD3BJ/6-311++G** level of theory in vacuum

S-ethyl 4-aminobenzene-1-sulfonothioate (**1**)

0 1			
C	2.41575400	-0.76394600	1.06012100
C	1.13957800	-0.23563200	1.17895500
C	0.62578400	0.55442900	0.15279500
C	1.37938300	0.82430700	-0.98922000
C	2.65211600	0.29528300	-1.10616100
C	3.19088000	-0.50932300	-0.08420800
H	2.82637700	-1.36677700	1.86251700
H	0.54695400	-0.41273100	2.06690800
H	0.96843800	1.45031600	-1.77095300
H	3.24743400	0.51208200	-1.98615800
N	4.48048300	-0.99470400	-0.18267800
H	4.73856700	-1.76272000	0.41619900
H	4.90095800	-1.02310200	-1.09785300
S	-1.01948900	1.21612600	0.28952800
O	-1.08256000	2.48604900	-0.43224200
O	-1.42165800	1.10626400	1.69437500
S	-2.23296100	-0.12044900	-0.87099700
C	-2.15911000	-1.58995100	0.23651400
H	-2.47019500	-1.26793600	1.22986500
H	-1.12480400	-1.93588000	0.27873300
C	-3.07759500	-2.67811600	-0.31275500
H	-3.03460700	-3.55374200	0.34028400
H	-4.11450000	-2.33827300	-0.35536200
H	-2.77578400	-2.98995300	-1.31523100

S-methyl 4-acetamidobenzene-1-sulfonothioate (**2**)

0 1			
C	-1.52949000	-0.76549600	-0.44368600
C	-0.15551900	-0.79837400	-0.64294900
C	0.61465500	0.32095200	-0.34445400
C	0.03870500	1.48703100	0.15326800
C	-1.33102100	1.52077800	0.35269600
C	-2.12574400	0.40051900	0.06003000
H	-2.14236200	-1.62169600	-0.67428600
H	0.31799100	-1.68723100	-1.03936300
H	0.65659400	2.34958500	0.36537700
H	-1.79377500	2.42472100	0.73441900
N	-3.50447600	0.52012200	0.28854700
H	-3.80250600	1.41565300	0.64320200
S	2.37886400	0.26098600	-0.58177400
O	2.88586300	1.63059100	-0.69913400
O	2.69832600	-0.72746400	-1.61562200
S	2.97394800	-0.55855700	1.32231000
C	4.71746200	-0.86975300	0.86216500
H	5.16755800	-1.36536600	1.72260100
H	5.23487700	0.06832000	0.66737600
C	-4.50315900	-0.41729400	0.09419400
C	-5.89538500	0.06714500	0.44999000
H	-6.30874900	-0.59888500	1.20957100
H	-5.92967100	1.09392300	0.81858900
H	-6.52608000	-0.01452600	-0.43720400
O	-4.30052500	-1.53947700	-0.32535900
H	4.76013500	-1.52644700	-0.00529000

S-ethyl 4-acetamidobenzene-1-sulfonothioate (**3**)

0 1			
C	-1.56952200	0.47384300	0.69792200
C	-0.19964000	0.35483700	0.90889900
C	0.51178700	-0.67056600	0.29637800
C	-0.12346800	-1.59676900	-0.52841900
C	-1.48531200	-1.48085500	-0.73901000
C	-2.22057900	-0.44650900	-0.13402400
H	-2.13495500	1.26095400	1.16986100
H	0.31324100	1.04356100	1.56751700
H	0.44391800	-2.39609000	-0.98706900
H	-1.99150900	-2.20058400	-1.37375300
N	-3.59795600	-0.40492300	-0.39827200
H	-3.94280400	-1.14629000	-0.98820200
S	2.27369700	-0.80611500	0.55823500
O	2.65453400	-2.21544900	0.48622300
O	2.60443400	0.01989900	1.72140500
S	3.11742200	0.05936400	-1.21288300
C	2.56792100	1.80814500	-1.02333500
H	1.50686300	1.79805700	-0.76462800
H	2.64069300	2.19697000	-2.04217700
C	3.39553300	2.63331400	-0.04674000
H	3.02230100	3.66217400	-0.02928700
H	3.33419500	2.22331200	0.96088300
H	4.44506500	2.65422200	-0.34669600
C	-4.53764500	0.51263200	0.03467400
C	-5.95005400	0.23771500	-0.44372000
H	-6.31563900	1.12252000	-0.96759400
H	-6.03714600	-0.62917200	-1.10117100
H	-6.58678000	0.08660400	0.43022300
O	-4.27194100	1.46449000	0.74219800

Table S2. Cartesian cordinates of thiosulfonates **1-3** calculated optimised at B3LYPGD3BJ/6-311++G** level of theory in water (PCM)

S-ethyl 4-aminobenzene-1-sulfonothioate (**1**)

0 1			
C	-2.33878300	0.07275500	1.18062300
C	-0.99488100	-0.24598400	1.25379500
C	-0.32551700	-0.67270800	0.10468600
C	-0.99168000	-0.76861200	-1.12096400
C	-2.33315000	-0.44971600	-1.19274300
C	-3.03410600	-0.02161400	-0.04282000
H	-2.86367000	0.39872600	2.07067400
H	-0.46409400	-0.16761800	2.19293700
H	-0.46099800	-1.08753800	-2.00891600
H	-2.85693000	-0.52953900	-2.13798800
N	-4.37325400	0.25464400	-0.10893300
H	-4.79832100	0.76789900	0.64769800
H	-4.79016300	0.40782900	-1.01410200
S	1.40590200	-1.00366800	0.17548900
O	1.74621500	-2.11561900	-0.72713500
O	1.79569700	-1.08446600	1.59303600
S	2.37238400	0.68097100	-0.72444200
C	2.07787433	1.96121320	0.57488347
H	2.45182317	1.49827941	1.48918929
H	0.99738493	2.06314950	0.68669460
C	2.76871231	3.28815523	0.29253100
H	2.56361319	3.98425409	1.11098214
H	3.85001191	3.15771257	0.21873155
H	2.40995997	3.73718550	-0.63363288

S-methyl 4-acetamidobenzene-1-sulfonothioate (**2**)

0 1			
C	-1.39499400	0.55186600	0.68316200
C	-0.02127300	0.53884800	0.89446300
C	0.75977600	-0.44470200	0.29589100
C	0.19374500	-1.42142200	-0.52453800
C	-1.17243100	-1.40870500	-0.73357700
C	-1.98106400	-0.42386200	-0.13629000
H	-2.01094600	1.30475400	1.14582200
H	0.43534100	1.28331200	1.53252800
H	0.81125400	-2.18105000	-0.98510700
H	-1.62488300	-2.16698600	-1.36185800
N	-3.35337400	-0.48618300	-0.40209900
H	-3.63811700	-1.24534400	-1.00385800
S	2.52001200	-0.43792900	0.54634700
O	3.02860000	-1.81551100	0.48637900
O	2.80752900	0.39425900	1.72330100
S	3.32721100	0.49855600	-1.19389400
C	5.07499210	0.61624656	-0.67169034
H	5.60292158	1.02453093	-1.53362972
H	5.46608126	-0.37114991	-0.43598612
C	-4.36354300	0.33811200	0.03819200
C	-5.74271500	-0.03294500	-0.45685600
H	-6.15391100	0.81688900	-1.00523500
H	-5.75619200	-0.91245500	-1.10015300
H	-6.38453100	-0.21273700	0.40782100
O	-4.17577400	1.29970400	0.77200100
H	5.17720655	1.29622976	0.17256176

S-ethyl 4-acetamidobenzene-1-sulfonothioate (**3**)

0 1			
C	-1.48434600	-0.12807800	0.93727600
C	-0.11805900	-0.29485800	1.12989900
C	0.67827400	-0.73403100	0.07779300
C	0.13599300	-1.00733900	-1.17846800
C	-1.22211100	-0.83999200	-1.37051900
C	-2.04669000	-0.39681800	-0.31930300
H	-2.11209200	0.20834400	1.74541500
H	0.32346900	-0.08634800	2.09484600
H	0.76562500	-1.34231800	-1.99216200
H	-1.65590100	-1.05167500	-2.34065100
N	-3.40860700	-0.25244400	-0.60315400
H	-3.67513100	-0.48547200	-1.54892900
S	2.43342400	-0.89175100	0.31466400
O	2.92741400	-2.06475700	-0.42162700
O	2.70175200	-0.77554000	1.75587100
S	3.30385700	0.76207500	-0.71413400
C	2.72312004	2.15981104	0.34689887
H	1.68425907	1.90627252	0.56270846
H	2.71172873	3.05383720	-0.27819799
C	3.53544818	2.33782887	1.62192054
H	3.11759689	3.16463155	2.20326018
H	3.50047444	1.43656929	2.23664175
H	4.57856291	2.56846272	1.40542442
C	-4.42928700	0.16908600	0.21876400
C	-5.78628000	0.23214900	-0.44405700
H	-6.07163400	1.28202000	-0.54734500
H	-5.81449200	-0.23761500	-1.42712200
H	-6.51567600	-0.24923700	0.20833900
O	-4.26307200	0.48420900	1.38961700

Table S3. Cartesian cordinates of thiosulfinates **1-3** calculated optimised at B3LYPGD3BJ/6-311++G** level of theory in vacuum

S-ethyl 4-aminobenzene-1-thiosulfinate (**1**)

0 1

C	-2.50659900	-1.27613900	-0.51107000
C	-1.16667900	-0.98673500	-0.71697100
C	-0.66322500	0.25467900	-0.33209300
C	-1.49363400	1.21361800	0.23660200
C	-2.83486400	0.92373500	0.44096500
C	-3.36089900	-0.32538100	0.07428100
H	-2.90212300	-2.24123400	-0.80835400
H	-0.51574300	-1.73076500	-1.16185000
H	-1.07689400	2.17558200	0.51035200
H	-3.48659800	1.66999100	0.88245900
N	-4.71308700	-0.59692800	0.23154400
H	-4.98107600	-1.56881200	0.24760400
H	-5.21126000	-0.03488600	0.90439500
S	1.06330700	0.68658400	-0.64909400
O	1.22693800	2.13610400	-0.28729500
S	1.82279000	-0.59010300	0.97022600
C	3.61287600	-0.30984400	0.64621700
H	4.10411800	-0.66044800	1.55657400
H	3.77242400	0.76908600	0.59192700
C	4.15005800	-1.02847700	-0.58559900
H	5.22078300	-0.83228200	-0.69928100
H	4.00294400	-2.10740400	-0.50466900
H	3.64977500	-0.68560100	-1.49371400

S-methyl 4-acetamidobenzene-1- thiosulfinate (**2**)

0 1

C	1.38731500	0.90170200	0.14128500
C	0.02023800	1.12941700	0.01432800
C	-0.80850900	0.13410700	-0.48321000
C	-0.29163400	-1.09640000	-0.88399600
C	1.06823600	-1.32763800	-0.75872200
C	1.91906400	-0.33535100	-0.24460600
H	2.04090500	1.66521000	0.53139600
H	-0.41227200	2.08122600	0.29870200
H	-0.94105400	-1.87037800	-1.27558200
H	1.47868300	-2.28532300	-1.06194900
N	3.28590300	-0.65189800	-0.15238100
H	3.53179800	-1.57726900	-0.46767100
S	-2.56966500	0.50976400	-0.70990100
O	-2.75779100	1.94929600	-0.33134500
S	-3.18936000	-0.82363500	0.91608000
C	-4.99047900	-0.66245200	0.63643700
H	-5.47368500	-1.31118000	1.36758100
H	-5.26202900	-0.99060100	-0.36723000
C	4.32823200	0.12609300	0.31084500
C	5.68513800	-0.55149500	0.26622100
H	6.09296600	-0.57410300	1.27853300
H	5.66350200	-1.56763100	-0.13209200
H	6.35477900	0.05687200	-0.34447200
O	4.19219300	1.26139600	0.72451400
H	-5.31134100	0.36497700	0.80374600

S-ethyl 4-acetamidobenzene-1-thiosulfinate (**3**)

0 1

C	1.73845300	0.90746700	0.07439200
C	0.37225700	1.10538000	-0.10369100
C	-0.43015500	0.06590200	-0.55148200
C	0.11385900	-1.18127800	-0.85306000
C	1.47280600	-1.38255500	-0.67805400
C	2.29664900	-0.34467200	-0.21185600
H	2.37102900	1.70567300	0.42798900
H	-0.08046300	2.06773800	0.10292900
H	-0.51501000	-1.99044000	-1.20465500
H	1.90378900	-2.35260400	-0.90364300
N	3.66459100	-0.63400600	-0.06277100
H	3.93070900	-1.57504000	-0.30732500
S	-2.18825800	0.40434300	-0.85198800
O	-2.39818100	1.86123800	-0.55567600
S	-2.85550200	-0.87151500	0.79681500
C	-4.64890500	-0.85397200	0.37788400
H	-5.05316000	-1.73070300	0.88913000
H	-4.74391800	-1.03490500	-0.69580000
C	-5.36814500	0.42168800	0.80224500
H	-6.43083300	0.35283400	0.54873500
H	-5.27995200	0.57691200	1.87939700
H	-4.94908400	1.29617300	0.30235100
C	4.68401000	0.18911600	0.37218200
C	6.05050300	-0.46956500	0.40814500
H	6.43511300	-0.41083600	1.42802400
H	6.05158300	-1.51264400	0.08619400
H	6.72597100	0.10170200	-0.23135900
O	4.52246300	1.34799600	0.70317300

Table S4. Cartesian cordinates of thiosulfinates **1-3** calculated optimised at B3LYPGD3BJ/6-311++G** level of theory in water (PCM)

S-ethyl 4-aminobenzene-1-thiosulfinate (**1**)

0 1			
C	-2.24659600	0.13809200	1.17338000
C	-0.91812600	-0.21497800	1.35166200
C	-0.18264600	-0.72263400	0.27879700
C	-0.77895800	-0.88923500	-0.97453800
C	-2.10444600	-0.54634500	-1.15324200
C	-2.86291800	-0.01940500	-0.08267600
H	-2.81765600	0.53372100	2.00504400
H	-0.45330800	-0.09080800	2.32302200
H	-0.19782300	-1.27913500	-1.80135900
H	-2.57182300	-0.67319300	-2.12309000
N	-4.19134600	0.28418800	-0.25766500
H	-4.63290600	0.88432500	0.42234400
H	-4.52674200	0.40770800	-1.20094600
S	1.52636400	-1.12567500	0.56162700
O	1.86179800	-2.34767600	-0.27237300
S	2.51877900	0.53248700	-0.54120900
C	4.28191622	0.11889625	-0.19266983
H	4.80782031	0.32579641	-1.12582182
H	4.34417743	-0.95989968	-0.02906664
C	4.87881297	0.90308467	0.96866841
H	5.92725950	0.61951109	1.10241692
H	4.83916425	1.97684343	0.77420703
H	4.34933762	0.70157166	1.89939316

S-methyl 4-acetamidobenzene-1- thiosulfinate (**2**)

0 1			
C	1.16547000	0.90734400	0.02434900
C	-0.20392900	1.09698700	-0.14012400
C	-0.99056700	0.07621400	-0.65442300
C	-0.42706500	-1.14298000	-1.03123500
C	0.93394100	-1.33558800	-0.87006700
C	1.74418000	-0.31578100	-0.33945600
H	1.78199800	1.69309900	0.42856800
H	-0.66383600	2.03797700	0.13366100
H	-1.04149900	-1.93963200	-1.43333000
H	1.37905000	-2.28156500	-1.15630000
N	3.11325300	-0.59659600	-0.21107300
H	3.39058400	-1.51589600	-0.52322400
S	-2.75837000	0.35760900	-0.90685400
O	-2.99618400	1.80482000	-0.52731500
S	-3.50688800	-0.93597300	0.69501500
C	-5.30376833	-0.77389886	0.41514869
H	-5.77565916	-1.51948224	1.05527731
H	-5.54132634	-0.99215346	-0.62537302
C	4.12036000	0.19515300	0.28259200
C	5.48905800	-0.44827200	0.28511300
H	5.82752400	-0.53736300	1.31982100
H	5.51211600	-1.43325000	-0.18082500
H	6.18437500	0.21167300	-0.23640500
O	3.94180900	1.33384700	0.69919400
H	-5.64677307	0.22178868	0.69012652

S-ethyl 4-acetamidobenzene-1-thiosulfinate (**3**)

0 1			
C	1.25302900	-0.50316500	-0.81899200
C	-0.10818600	-0.77910100	-0.91287300
C	-0.88108900	-0.85976400	0.23652300
C	-0.31051600	-0.69614200	1.49865100
C	1.04288700	-0.42602500	1.59701700
C	1.83751700	-0.31846500	0.44115100
H	1.85909600	-0.42923700	-1.70680400
H	-0.57193500	-0.92853600	-1.87952200
H	-0.91527400	-0.76045100	2.39521800
H	1.49351300	-0.29182300	2.57372000
N	3.19833500	-0.03468900	0.63187200
H	3.48425500	0.05494700	1.59597100
S	-2.64577200	-1.22049800	0.09857800
O	-2.87877200	-1.49863700	-1.37265200
S	-3.43687000	0.76918800	0.56812200
C	-5.24192413	0.40710077	0.66101427
H	-5.59076197	0.96733527	1.52964377
H	-5.34004768	-0.65309848	0.90769651
C	-6.01457665	0.78522813	-0.59579528
H	-7.07601566	0.56194968	-0.45168562
H	-5.91838837	1.85190506	-0.80868448
H	-5.66404763	0.22714965	-1.46334769
C	4.18875900	0.14299000	-0.30223300
C	5.55649200	0.43402100	0.27367500
H	5.91957500	1.37216300	-0.15007100
H	5.56800400	0.50482600	1.36116100
H	6.23985200	-0.35851400	-0.03851600
O	3.99837300	0.07189300	-1.51076600
